# Changes in Metabolic Syndrome Status and Breast Cancer Risk: A Nationwide Cohort Study

**DOI:** 10.3390/cancers13051177

**Published:** 2021-03-09

**Authors:** In Young Choi, Sohyun Chun, Dong Wook Shin, Kyungdo Han, Keun Hye Jeon, Jonghan Yu, Byung Joo Chae, Mina Suh, Yong-Moon Park

**Affiliations:** 1 Total Healthcare Center, Kangbuk Samsung Hospital, Sungkyunkwan University School of Medicine, Seoul 04514, Korea; inyoungb.choi@samsung.com; 2 Department of Family Medicine, Samsung Medical Center, Sungkyunkwan University School of Medicine, Seoul 06351, Korea; kh1228.jeon@samsung.com; 3 International Healthcare Center, Samsung Medical Center, Seoul 06351, Korea; 4 Department of Clinical Research Design & Evaluation, Samsung Advanced Institute for Health Science & Technology (SAIHST), Sungkyunkwan University, Seoul 06355, Korea; 5 Department of Digital Health, Samsung Advanced Institute for Health Science & Technology (SAIHST), Sungkyunkwan University, Seoul 06355, Korea; 6 Department of Statistics and Actuarial Science, Soongsil University, Seoul 06978, Korea; hkd@ssu.ac.kr; 7 Division of Breast and Endocrine Surgery, Department of General Surgery, Samsung Medical Center, Sungkyunkwan University School of Medicine, Seoul 06351, Korea; jonghan.yu@samsung.com; 8 National Cancer Control Institute, National Cancer Center, Goyang 10408, Korea; bj.chae@samsung.com (B.J.C.); omnibus@ncc.re.kr (M.S.); 9 Graduate School of Cancer Science and Policy, National Cancer Center, Goyang 10408, Korea; 10 Department of Epidemiology, Fay W. Boozman College of Public Health, University of Arkansas for Medical Sciences, Little Rock, AR 72205, USA; ypark@uams.edu

**Keywords:** breast cancer, metabolic syndrome, components, changes, postmenopausal

## Abstract

**Simple Summary:**

There is also growing evidence for metabolic syndrome as a risk factor for BC. However, no studies have yet looked at how the risk of developing breast cancer varies with changes in metabolic syndrome status. It is important to identify the risk of BC among women who develop or recover from metabolic syndrome in disease prevention. Therefore, this study aimed to investigate the association between changes in metabolic syndrome and subsequent breast cancer occurrence. As a result, the risk of breast cancer differed significantly according to the changes of metabolic syndrome status. Individuals who improved from metabolic syndrome had the same risk of breast cancer as those who were sustained non-metabolic syndrome. Thus, efforts to improve metabolic syndrome may provide an added benefit of a reduced risk of breast cancer.

**Abstract:**

Objective: To our knowledge, no studies have yet looked at how the risk of developing breast cancer (BC) varies with changes in metabolic syndrome (MetS) status. This study aimed to investigate the association between changes in MetS and subsequent BC occurrence. Research Design and Methods: We enrolled 930,055 postmenopausal women aged 40–74 years who participated in a biennial National Health Screening Program in 2009–2010 and 2011–2012. Participants were categorized into four groups according to change in MetS status during the two-year interval screening: sustained non-MetS, transition to MetS, transition to non-MetS, and sustained MetS. We calculated multivariable-adjusted hazard ratios (aHRs) and 95% confidence intervals (CIs) for BC incidence using the Cox proportional hazards models. Results: At baseline, MetS was associated with a significantly increased risk of BC (aHR 1.11, 95% CI 1.06–1.17) and so were all of its components. The risk of BC increased as the number of the components increased (aHR 1.46, 95% CI 1.26–1.61 for women with all five components). Compared to the sustained non-MetS group, the aHR (95% CI) for BC was 1.11 (1.04–1.19) in the transition to MetS group, 1.05 (0.96–1.14) in the transition to non-MetS group, and 1.18 (1.12–1.25) in the sustained MetS group. Conclusions: Significantly increased BC risk was observed in the sustained MetS and transition to MetS groups. These findings are clinically meaningful in that efforts to recover from MetS may lead to reduced risk of BC.

## 1. Introduction

Breast cancer (BC) is the most frequent malignancy in women as well as the leading cause of mortality among women with cancer [1,2]. The cancer incidence and cancer-related mortality rates of BC are increasing rapidly worldwide [3]. In the US, the estimated total BC cases and BC-related deaths increased from 194,280 and 40,610, respectively in 2009, to 268,670 and 41,400, respectively in 2018 [4]. In Korea, there were 22,300 new BC cases in 2017 [5], and the incidence rate of BC is expected to increase during the following 10 years in Korea, owing to an increase in the aging population and the adoption of a Westernized lifestyle [6].

A ‘common soil’ hypothesis has been proposed as underlying the metabolic syndrome (MetS) characterized by insulin resistance and chronic inflammation, which may also be relevant in the pathophysiology of BC and its progression [7,8]. There is also growing evidence for MetS as a risk factor for BC. Despite some inconsistencies in results in earlier studies [8,9,10,11], recent large meta-analyses including those prior studies agreed on the association between metabolic syndrome and increased risk of breast cancer, especially in postmenopausal women [12,13,14]. In a meta-analysis of nine independent cohorts and 97,277 women, a positive association was observed between MetS and BC risk (adjusted risk ratio (aRR) 1.47, 95% CI 1.15–1.87) [13]. Similarly, a recent meta-analysis of 17 cohort studies and 602,195 women observed an association between MetS and increased BC risk (aRR 1.15, 95% CI 1.05–1.26) [14]. 

The studies above have found that MetS may be an important predictor of BC. In addition, weight control and physical activity, which are effective ways to modulate personal metabolic status [15,16], have proven to be effective in reducing the risk of BC [17]. However, to our knowledge, no studies have yet looked at how the risk of developing BC varies with changes in MetS status. It is important to identify the risk of BC among women who develop or recover from MetS in disease prevention. Furthermore, comparing the different risks of BC associated with changes of each MetS component would reveal a potential approach for decreasing MetS-related BC incidences. Therefore, this study aimed to investigate the association between changes in MetS and subsequent BC occurrence. 

## 2. Materials and Methods

### 2.1. Data Source

The Korean National Health Insurance Service (NHIS) provides the single and mandatory national health insurance that covers comprehensive medical care to all Koreans. The NHIS also offers a biennial health examination concentrating on cardiovascular disease for all individuals aged 40 years and above and all employees of any age [18]. The examination includes anthropometric measurements (height, weight, waist circumference (WC), and blood pressure), a questionnaire on lifestyle behaviors (smoking, alcohol consumption, and physical activity), and laboratory tests (glucose and lipid profiles, etc.). BC screening is provided for all women aged 40 years and above [19]. Women participants are required to provide their reproductive history before BC screening examination.

The NHIS has built a National Health Information Database (NHID), a comprehensive health database, for all Koreans. This includes health data on eligibility (age, sex, income level, etc.), diseases such as diagnosis date and codes according to the International Classification of Disease 10th revision (ICD-10), information of medical care (tests, treatments, etc. based on the medical expense that medical service providers charge for their medical expenditures), prescription information, and health examinations (results of cardiovascular and cancer screening) [20,21]. Many previous epidemiological studies have used this database and its details have been described elsewhere [22,23]. 

The Institutional Review Board of the Samsung Medical Centre (IRB File No. SMC 2020-05-065) approved this study. They waived the requirement for informed consent because the raw data from the NHIS of Korea were anonymized.

### 2.2. Study Population

**Figure 1:**
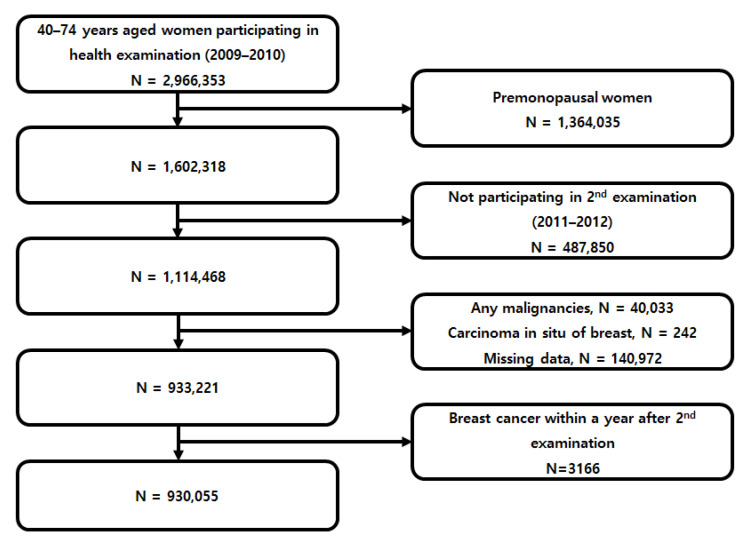
Patient flow chart.

Data from the NHIS database were extracted for women aged 40–74 years who had undergone two consecutive national health examinations in 2009–2010 (first) and 2011–2012 (second) to determine change in metabolic syndrome status. The first examination data from 1 January 2009 to 31 December 2010 were considered as the baseline. Among 2,966,353 women who participated the first examination, those excluded from this study were women who were premenopausal (*n* = 1,364,035), who did not participate in a follow-up examination from 1 January 2011 to 31 December 2012 (*n* = 487,850), who were diagnosed with any malignancies (*n* = 40,033) or carcinoma in situ of the breast (ICD-10 code: D05) before the date of the 2nd examination (*n* = 242), and who had missing data (*n* = 140,972). As subjects who developed BC immediately after health examination may have an unclear temporal relationship with the MetS status identified at the health examination, we gave a 1-year lag time and further excluded 3166 women diagnosed with BC within a year after their second health examination. Finally, a total of 930,055 individuals were included in the analysis for baseline (Figure 1). 

### 2.3. Independent Variables

MetS was determined based on anthropometric data and laboratory results. WC was measured at the midpoint of the abdomen between the lower margin of the last palpable rib and the top of the iliac crest [24]. Participants’ brachial blood pressure was measured with their arm in the appropriate position after sitting and resting for five minutes. Blood samples for laboratory tests were collected after overnight fasting. The definition of MetS followed the 2009 agreement of the International Diabetes Federation and American Heart Association/National Heart, Lung, and Blood Institute [25]. By this definition, participants were defined to have MetS if three or more of the following criteria were true: (1) serum triglycerides ≥ 150 mg/dL or patient was taking lipid-lowering medication; (2) serum high-density lipoprotein (HDL) cholesterol < 50 mg/dL or use of lipid-lowering medication; (3) systolic blood pressure ≥ 130 mmHg, diastolic blood pressure ≥ 85 mmHg, or use of antihypertensive medication; (4) fasting plasma glucose ≥ 100 mg/dL or use of hypoglycemic agents; (5) patient was diagnosed with abdominal obesity. Abdominal obesity was defined as a waist circumference of 85 cm or more, according to the definition from the Korean Society for the Study of Obesity [26].

This study compared 2009–2010 and 2011–2012 results of the national health screening program [22,23]. Using changes in MetS status during the continuous biennial checkup, participants were categorized into four groups: sustained non-MetS, transition to MetS, transition to non-MetS, and sustained MetS.

### 2.4. Covariates 

Previously known risk factors of BC—age, hormone therapy, age at menarche, age at menopause, smoking, alcohol consumption, exercise, and income status—were included in the analysis. Information on reproductive factors and health-related behaviors was obtained using a self-administered questionnaire. Hormone replacement therapy (HRT) was divided into categorical subgroups: never, <2 years, 2–5 years, ≥5 years, and unknown. Health-related behaviors included histories of smoking, alcohol consumption, and regular exercise. Smoking history was classified as: never, former, and current smoker. Alcohol consumption was divided into three levels: non, mild-to-moderate (<30 g of alcohol/day), and heavy (≥30 g/day). Regular physical activity was defined as moderate physical activity for more than 30 min daily and more than five days per week over the past week. Income status was divided into quartiles based on the amount of health insurance premiums paid (Korean premiums are determined by income level), where those who received medical aid (the poorest 3%) were merged with the lowest income quartile.

### 2.5. Outcome Variable and Follow-Up

Incidence of BC, defined by the new diagnosis code of C50 (malignant neoplasm of the breast, i.e., invasive BC) and D05 (carcinoma in situ of the breast) from a special co-payment reduction program in Korea, was the primary endpoint according to the ICD-10 codes. The NHIS operates a special co-payment reduction program for cancer patients to relieve their significant financial burden. Only 5% of the total medical expenses for diagnostic tests or treatments for cancer is charged to cancer patients. The physician must have sufficient evidence of diagnosis to enroll in this program, and almost all patients apply for this program because of the economic benefit. Thus, the definition of incidence of BC by the newly registered C50 and D05 codes from the program is fairly reliable. Also, this has been widely used in previous studies [23,27,28]. The cohort was followed to the date of incidence of BC, death, or the last day of the study (31 December 2018), whichever came first. 

### 2.6. Statistical Analyses

Continuous variables were expressed as means ± standard deviation and categorical variables as numbers and percentages. The incidence rates for BC were calculated by dividing the total follow-up period by the number of incident cases and presented per 1000 person-years. The Cox proportional hazards model evaluated hazard ratios (HRs) and 95% confidence interval (CI) values for BC. A multivariable-adjusted proportional hazards model was applied: (1) Model 1 was adjusted for age; (2) Model 2 was further adjusted for age, smoking, alcohol consumption, and physical activity; (3) Model 3 was adjusted for income status, age at menarche, age at menopause, and HRT in addition to Model 2. We also performed the same analysis with invasive BC and carcinoma in situ as separate outcomes. Statistical analyses were performed using Statistical Analysis System (SAS) version 9.4 (SAS Institute Inc., Cary, NC, USA), and a *p* value less than 0.05 was considered statistically significant.

## 3. Results

### 3.1. Characteristics of the Participants

During the mean 6.40 years of follow-up (max 8.0 years), a total of 6844 cases of BC were observed among the total 930,055 participants. In Table 1, the BC group is compared to those without BC. Compared with the group without BC, the group with BC was slightly younger, had higher BMI and WC, and included more smokers and those who do regular physical activity. Dyslipidemia was more prevalent, menarche age was younger, menopause age was older, and HRT experience was more common in the BC group. There was no significant difference in the percentages of alcohol consumption, hypertension, diabetes mellitus, blood pressure, glucose, total cholesterol, or HDL cholesterol levels between the two groups. 

### 3.2. The Risk of BC and Basal MetS

Having MetS was associated with a significantly increased risk of BC (adjusted hazard ratio (aHR) 1.11, 95% confidence interval (CI) 1.06–1.17), after adjustment for covariates (Model 3) in Table 2. When analyzed by components of the MetS, central obesity (aHR 1.15, 95% CI 1.09–1.21), glucose intolerance (aHR 1.07, 95% CI 1.02–1.13), high blood pressure (aHR 1.13, 95% CI 1.08–1.19), high triglycerides (aHR 1.08, 95% CI 1.03–1.13), and low HDL cholesterol (aHR 1.06, 95% CI 1.01–1.11) were significantly associated with an increased risk of BC. When looking at the risk of BC according to the number of MetS components with which a patient was diagnosed, there was a trend that the risk of BC increased as the number increased (aHR 1.43, 95% CI 1.26–1.61 for women with all five components, *p* for trends <0.001). The results analyzing only invasive BC as an outcome while censoring carcinoma in situ were similar to the main results (Supplementary Table S1). 

### 3.3. The Risk of BC based on Changes of MetS Status

The risk of BC based on changes of MetS and its components are represented in Table 3. Compared to the sustained non-MetS group, aHR and 95% CIs for BC was 1.11 (1.04–1.19) in the transition to MetS group and 1.18 (1.12–1.25) in the sustained MetS group. In addition, the sustained groups in each component of MetS showed the highest risks: aHRs (95% CI) were 1.23 (1.16–1.31), 1.14 (1.08–1.21), 1.13 (1.07–1.20), 1.13 (1.07–1.20), and 1.10 (1.04–1.16) for waist circumference, fasting glucose, blood pressure, triglycerides, and HDL cholesterol, respectively. On the other hand, the transition to non-MetS group had lower risk of BC than the sustained MetS group (*p* < 0.05), and the risk was close to that of the sustained non-MetS group (aHR 1.05, 95% CI 0.96–1.14. The same pattern was displayed for each MetS component: aHRs (95% CI) were 1.06 (0.97–1.15), 0.95 (0.89–1.03), 1.00 (0.92–1.10), 1.03 (0.95–1.12), and 1.01 (0.94–1.10) for waist circumference, fasting glucose, blood pressure, triglycerides, and HDL cholesterol, respectively. The results analyzing only invasive BC as an outcome while censoring DCIS were similar to the main results (Supplementary Table S2). 

## 4. Discussion

To the best of our knowledge, this is the first study to examine the different BC risks according to MetS status changes. In this study, the risk of BC differed significantly according to the changes of MetS status. Individuals who improved from MetS had the same risk of BC as those who were sustained non-MetS. Of the four groups, the group with sustained MetS at both baseline and in the subsequent examination had the highest risk of BC. Thus, efforts to improve MetS may provide an added benefit of a reduced risk of BC.

There are three main pathophysiological explanations for MetS that pose an increased risk of BC: insulin resistance, chronic low-grade inflammation, and adipose tissue estrogen production [7]. Insulin was found to stimulate insulin–IGF-1 signaling in tumor cells and result in an activation of the oncogenic Ras–MAPK and PI3K–Akt pathways (MAPK = mitogen-activated protein kinase, PI3K = phosphoinositide 3-kinase), which subsequently stimulate tumor cell growth [29]. Expansion of adipose tissue in obesity induces an increased release of proinflammatory proteins that may exert mitogenic, angiogenic, and antiapoptotic actions in tumor progression. As adipose tissue mainly produces estrogens via the enzyme aromatase in postmenopausal women, obese postmenopausal women usually exhibit elevated estrogen levels and are at risk of developing estrogen-dependent BC.

Our study showed that having MetS was associated with an 11% increase in risk of BC. This is consistent with previous studies. Guo’s meta-analysis of 17 follow-up studies with 602,195 women concluded that postmenopausal women with MetS were associated with a significantly increased risk of BC incidence (adjusted risk ratio = 1.25, 95% CI 1.12–1.39) [14]. According to a recent Korean cohort study of 13,377,349 women using NHIS, MetS increased the risk of all BC types (HR 1.15, 95% CI 1.12–1.17) in women aged >50 years [30].

However, whether improvement in MetS status actually changes the incidence of BC has been unclear. In this study, the risk of BC was not different in the transition to non-MetS group compared to the sustained non-MetS group, but was slightly lower than the sustained MetS group. Furthermore, all of the improvements among each component of the metabolic syndrome exhibited the same risk of BC that remained normal in that component. In addition, the transition to the MetS group as well as the sustained MetS group showed increased risks of BC compared to the sustained non-MetS group (aHR 1.11, 95% CI 1.04–1.19). Although it is not possible to prove causality in this observational study, our results imply that efforts to improve MetS and its components may be an effective strategy to prevent BC.

Lifestyle factors such as diet, physical activity, smoking, and alcohol consumption can strongly influence metabolic parameters such as waist circumference, blood pressure, fasting glucose, triglycerides, and HDL cholesterol [31,32,33]. Previous studies have also shown that lifestyle modifications, which include maintaining a healthy weight, limiting alcohol consumption, smoking cessation, and being physically active on a regular basis, are substantially associated with a reduced risk of developing BC [34,35,36]. The present longitudinal study indicated that changes in MetS status remained independent determinants of BC risk even after adjusting for lifestyle factors.

The natural history of DCIS is not completely understood despite potentially being on the pathway to invasive BC. Invasive BC and DCIS share many risk factors, but studies have also shown some differences, such as obesity [37,38]. Thus, in Supplementary Tables S1 and S2, we analyzed only invasive BC as an outcome while censoring DCIS, but the results were not significantly different from the analyses of total BC.

This study has several limitations. First, the duration of the two examination points where the MetS status was evaluated was relatively short, at 2 years. Longer, sustained changes might be associated with further increases in risk of BC. Second, this study could not include some risk factors for BC, such as BC family history, owing to the high rate of missing information in the database. Third, the mean follow-up was relatively short. Fourth, the interval between each screening was too short and the duration of MetS was not considered, which could influence BC occurrence by estrogen exposure and chronic inflammation. Lastly, since this study is an observational study, it is unclear whether this is a causal effect even though there was an association between MetS change and the risk of BC. Nevertheless, this study included a large cohort representing the national population and fully considered traditional BC risk factors.

## 5. Conclusions

In conclusion, this study demonstrated a significant association of changes in MetS status and its components with the risk of BC. These findings are clinically meaningful in that efforts to recover from MetS have the potential to reduce the risk of BC. A future prospective study is necessary to confirm whether interventions to improve MetS could prevent BC.

## Figures and Tables

**Table 1:** Characteristics of participants.

	Breast Cancer		
	Yes	No	*p*-Value
Number of participants	6844	923,211	
Age	59.0 ± 6.5	60.3 ± 7.0	<0.0001
Body mass index (kg/m^2^)	24.5 ± 3.1	24.2 ± 3.1	<0.0001
Waist circumference (cm)	80.2 ± 8.1	79.9 ± 8.5	0.0199
Smoking history			0.0292
Never	6606 (96.52)	893,906 (96.83)	
Former	87 (1.27)	8834 (0.96)	
Current	151 (2.21)	20,471 (2.22)	
Alcohol consumption			0.0852
None	5910 (86.35)	805,477 (87.25)	
Mild-to-moderate	900 (13.15)	113,300 (12.27)	
Heavy	34 (0.50)	4434 (0.48)	
Physical activity	1419 (20.73)	179,330 (19.42)	0.0064
Comorbidities			
Hypertension	3053 (44.61)	408,484 (44.25)	0.5476
Diabetes mellitus	852 (12.45)	112,937 (12.23)	0.5873
Dyslipidemia	2463 (35.99)	320,072 (34.67)	0.0224
Age at menarche	16.1 ± 1.8	16.4 ± 1.8	<0.0001
Age at menopause	50.6 ± 3.8	50.1 ± 3.9	<0.0001
HRT			<0.0001
Never	5076 (74.17)	730,674 (79.14)	
Less than 2 years	758 (11.08)	92,850 (10.06)	
2–5 years	392 (5.73)	38,692 (4.19)	
More than 5 years	383 (5.60)	29,688 (3.22)	
Do not know	235 (3.43)	31,307 (3.39)	
Bottom 3% income	1605 (23.45)	215,670 (23.36)	0.8603
Blood pressure (mmHg)			
Systolic	124.9 ± 15.9	125.0 ± 15.9	0.4753
Diastolic	76.6 ± 10.1	76.7 ± 10.1	0.4183
Laboratory findings (mg/dL)			
Glucose	99.1 ± 22.8	99.1 ± 23.0	0.8546
Total cholesterol	208.2 ± 44.8	208.4 ± 43.3	0.737
HDL cholesterol	58.2 ± 37.4	57.9 ± 35.3	0.5449
Triglycerides *	113.3 (111.9, 114.7)	114.4 (114.3, 114.6)	0.0115

Abbreviations: HRT = hormone replacement therapy, HDL = high-density lipoprotein. Data are presented as means ± standard deviation (SD) or proportions (%). * Geometric means (95% confidence interval).

**Table 2:** Risk of breast cancer incidence according to baseline metabolic syndrome status and its components.

	Number of Participants	Number of Breast Cancer Cases	Person-Years (P-Y)	Rate (Per 1000 P-Y)	Model 1 *	Model 2 ^†^	Model 3 ^‡^
**Metabolic syndrome ^§^**							
No	596,264	4759	3,815,960.9	1.25	1 (Ref.)	1 (Ref.)	1 (Ref.)
Yes	333,791	2636	2,130,328.8	1.24	1.10 (1.04, 1.15)	1.10 (1.05, 1.15)	1.11 (1.06, 1.17)
**Individual components ^§^**							
**Waist circumference**							
No	677,776	5348	4,334,934.3	1.23	1 (Ref.)	1 (Ref.)	1 (Ref.)
Yes	252,279	2047	1,611,355.4	1.27	1.12 (1.06, 1.18)	1.12 (1.07, 1.18)	1.15 (1.09, 1.21)
**Fasting glucose**							
No	596,665	4730	3,821,633.4	1.24	1 (Ref.)	1 (Ref.)	1 (Ref.)
Yes	333,390	2665	2,124,656.3	1.25	1.06 (1.01, 1.12)	1.06 (1.02, 1.12)	1.07 (1.02, 1.13)
**Blood pressure**							
No	393,351	3131	2,520,229.0	1.24	1 (Ref.)	1 (Ref.)	1 (Ref.)
Yes	536,704	4264	3,426,060.7	1.24	1.13 (1.08, 1.18)	1.13 (1.08, 1.18)	1.13 (1.08, 1.19)
**Triglycerides**							
No	582,539	4612	3,724,540.1	1.24	1 (Ref.)	1 (Ref.)	1 (Ref.)
Yes	347,516	2783	2,221,749.6	1.25	1.07 (1.02, 1.12)	1.07 (1.02, 1.12)	1.08 (1.03, 1.13)
**HDL cholesterol**							
No	530,770	4206	3,393,404.4	1.24	1 (Ref.)	1 (Ref.)	1 (Ref.)
Yes	399,285	3189	2,552,885.2	1.25	1.06 (1.01, 1.11)	1.06 (1.01, 1.11)	1.06 (1.01, 1.11)
**Number of components**							
0	144,848	1160	927,582.2	1.25	1 (Ref.)	1 (Ref.)	1 (Ref.)
1	226,443	1776	1,449,657.9	1.23	1.05 (0.98, 1.13)	1.05 (0.98, 1.13)	1.05 (0.97, 1.13)
2	224,973	1823	1,438,720.7	1.27	1.14 (1.06, 1.23)	1.14 (1.06, 1.23)	1.15 (1.07, 1.24)
3	182,378	1373	1,165,700.3	1.18	1.10 (1.02, 1.20)	1.11 (1.02, 1.20)	1.12 (1.03, 1.21)
4	111,414	908	710,635.6	1.28	1.23 (1.13, 1.35)	1.23 (1.13, 1.35)	1.25 (1.15, 1.37)
5	39,999	355	253,992.9	1.40	1.39 (1.23, 1.56)	1.39 (1.23, 1.57)	1.43 (1.26, 1.61)
*p*-value					<0.001	<0.001	<0.001

Abbreviations: Ref. = reference; HDL = high-density lipoprotein. * Model 1: adjusted for age. **^†^** Model 2: Model 1 + smoking, alcohol consumption, and physical activity. **^‡^** Model 3: Model 2 + duration of HRT, age at menarche, age at menopause, and income status. **^§^** Metabolic syndrome and components were defined from blood tests and anthropometric measurements from the 2009–2010 examinations: waist circumference ≥ 85 cm, systolic blood pressure ≥ 130 mmHg, diastolic blood pressure ≥ 85 mmHg or use of antihypertensive medications, fasting glucose ≥ 100 mg/dL or use of hypoglycemic agents, triglycerides ≥ 150 mg/dL or use of lipid-lowering medications, HDL cholesterol < 50 mg/dL or use of lipid-lowering medications. The presence of three or more out of five components was regarded as metabolic syndrome.

**Table 3:** Risk of breast cancer incidence according to changes in metabolic syndrome and its components.

		Number of Participants	Number of Breast Cancer Cases	Duration	Rate	Model 1 *	Model 2 ^†^	Model 3 ^‡^
**Metabolic Syndrome Status ^§^**								
No MetS–No MetS		431,790	3429	2,762,581.6	1.24	1 (Ref.)	1 (Ref.)	1 (Ref.)
No MetS–MetS		164,474	1330	1,053,379.3	1.26	1.11 (1.04, 1.18)	1.11 (1.04, 1.18)	1.11 (1.04, 1.19)
MetS–No MetS		79,687	595	509,303.6	1.17	1.03 (0.94,1.12)	1.03 (0.94,1.12)	1.05 (0.96, 1.14)
MetS–MetS		254,104	2041	1,621,025.2	1.26	1.17 (1.10, 1.23) **	1.17 (1.10, 1.24) **	1.18 (1.12, 1.25) **
**Metabolic Syndrome Components ^§^**								
**Waist Circumference** (≥85 cm)	No–No	588,701	4620	3,764,454.1	1.23	1 (Ref.)	1 (Ref.)	1 (Ref.)
	No–Yes	89,075	728	570,480.1	1.28	1.11 (1.03, 1.20)	1.11 (1.03, 1.20)	1.13 (1.04, 1.22)
	Yes–No	86,298	645	551,433.3	1.17	1.03 (0.95, 1.12)	1.04 (0.95, 1.13)	1.06 (0.97, 1.15)
	Yes–Yes	165,981	1402	1,059,922.1	1.32	1.19 (1.12, 1.27) **	1.20 (1.13, 1.27) **	1.23 (1.16, 1.31) **
**Fasting Glucose** (≥100 mg/dL)	No–No	474,211	3782	3,038,048.5	1.24	1 (Ref.)	1 (Ref.)	1 (Ref.)
	No–Yes	122,454	948	783,584.9	1.21	1.00 (0.94, 1.08)	1.01 (0.94, 1.08)	1.02 (0.95, 1.09)
	Yes–No	110,767	813	708,623.8	1.15	0.94 (0.88, 1.02)	0.95 (0.88,1.02)	0.95 (0.89, 1.03)
	Yes–Yes	222,623	1852	1,416,032.5	1.31	1.13 (1.07, 1.20) **	1.13 (1.07, 1.20) **	1.14 (1.08, 1.21) **
**Blood Pressure** (Systolic ≥130 or diastolic ≥85 mmHg)	No–No	260,587	2179	1,669,554.1	1.31	1 (Ref.)	1 (Ref.)	1 (Ref.)
	No–Yes	132,764	952	850,674.9	1.12	0.93 (0.86, 1.00)	0.93 (0.86, 1.00)	0.94 (0.87, 1.02)
	Yes–No	73,625	571	471718.6	1.21	0.99 (0.90, 1.09)	0.99 (0.91, 1.09)	1.00 (0.92, 1.10)
	Yes–Yes	463,079	3693	2,954,342.1	1.25	1.12 (1.06, 1.19) **	1.12 (1.06, 1.19) **	1.13 (1.07, 1.20) **
**Triglycerides** (≥150 mg/dL)	No–No	398,941	3146	2,548,932.5	1.23	1 (Ref.)	1 (Ref.)	1 (Ref.)
	No–Yes	183,598	1466	1,175,607.6	1.25	1.07 (1.00, 1.14)	1.07 (1.00, 1.14)	1.07 (1.00, 1.14)
	Yes–No	91,795	700	586,557.3	1.19	1.01 (0.93, 1.10)	1.01 (0.93, 1.10)	1.03 (0.95, 1.12)
	Yes–Yes	255,721	2083	1,635,192.3	1.27	1.12 (1.06, 1.19) **	1.12 (1.06, 1.19) **	1.13 (1.07, 1.20) **
**HDL cholesterol** (<50 mg/dL)	No–No	328,992	2619	2,101,628.0	1.25	1 (Ref.)	1 (Ref.)	1 (Ref.)
	No–Yes	201,778	1,587	1,291,776.4	1.23	1.05 (0.98, 1.12)	1.05 (0.98, 1.12)	1.04 (0.98, 1.11)
	Yes–No	108,086	828	691,023.8	1.20	1.01 (0.93, 1.09)	1.01 (0.93, 1.09)	1.01 (0.94, 1.10)
	Yes–Yes	291,199	2361	1,861,861.5	1.27	1.11 (1.05, 1.17) **	1.11 (1.05, 1.17) **	1.10 (1.04, 1.16) **

Abbreviations: Ref. = reference; HDL = high-density lipoprotein. * Model 1: adjusted for age. **^†^** Model 2: Model 1 + smoking, alcohol consumption, and physical activity. **^‡^** Model 3: Model 2 + duration of HRT, age at menarche, age at menopause, and income status. ^§^ Metabolic syndrome and components were defined from blood tests and anthropometric measurements from 2009–2010 and 2011–2012 examinations: waist circumference ≥ 85 cm, systolic blood pressure ≥ 130 mmHg, diastolic blood pressure ≥ 85 mmHg or use of antihypertensive medications, fasting glucose ≥ 100 mg/dL or use of hypoglycemic agents, triglycerides ≥ 150 mg/dL or use of lipid-lowering medications, HDL cholesterol < 50 mg/dL or use of lipid-lowering medications. The presence of three or more out of five components was regarded as metabolic syndrome. ** Difference statistically significant (*p* < 0.05) compared to the MetS–No MetS group or the Yes–No Group.

## Data Availability

Restrictions apply to the availability of these data. Data were obtained from Korea NHIS and are available at https://nhiss.nhis.or.kr/bd/ay/bdaya001iv.do (accessed on 10 January 2021) with the permission of Korea NHIS.

## Supplementary Materials

The following are available online at https://www.mdpi.com/2072-6694/13/5/1177/s1, Table S1: Risk of Invasive Breast Cancer According to Baseline Metabolic Syndrome Status and Its Components, Table S2: Risk of Invasive Breast Cancer According to Changes in Metabolic Syndrome and Its Components.
