# Supplementary material for: Changes in Metabolic Syndrome Status and Breast Cancer Risk: A Nationwide Cohort Study
Source: Cancers (Basel). 2021 Mar 9;13(5):1177. doi: 10.3390/cancers13051177 (PMC7967214; doi:10.3390/cancers13051177)
Supplement: Supplementary file 1 [file cancers-13-01177-s001.pdf]

Supplementary Materials: Changes in Metabolic Syndrome Status and Breast Cancer Risk: A Nationwide Cohort Study

In Young Choi, Sohyun Chun, Dong Wook Shin, Kyungdo Han, Keun Hye Jeon, Jonghan Yu, Byung Joo Chae, Mina Suh and Yong-Moon Park

Table S1. Risk of Invasive Breast Cancer According to Baseline Metabolic Syndrome Status and Its Components

	Number of Participants	Number of breast cancers	Person-year (P-Y)	Rate (Per 1,000 P-Y)	Model 1*	Model 2†	Model 3‡
**Metabolic syndrome§**							
No	596264	3881	3815960.85	1.02	1 (Ref.)	1 (Ref.)	1 (Ref.)
Yes	333791	2157	2130328.78	1.01	1.09 (1.04, 1.15)	1.09 (1.04, 1.15)	1.11 (1.05, 1.17)
**Individual components§**							
**Waist circumference**							
No	677776	4340	4334934.26	1.00	1 (Ref.)	1 (Ref.)	1 (Ref.)
Yes	252279	1698	1611355.37	1.05	1.14 (1.08, 1.21)	1.14 (1.08, 1.21)	1.17 (1.10, 1.24)
**Fasting glucose**							
No	596665	3879	3821633.36	1.02	1 (Ref.)	1 (Ref.)	1 (Ref.)
Yes	333390	2159	2124656.27	1.02	1.05 (0.99, 1.10)	1.05 (0.99, 1.10)	1.06 (1.00, 1.11)
**Blood pressure**							
No	393351	2502	2520228.96	0.99	1 (Ref.)	1 (Ref.)	1 (Ref.)
Yes	536704	3536	3426060.67	1.03	1.16 (1.10, 1.23)	1.17 (1.11, 1.23)	1.17 (1.11, 1.23)
**Triglycerides**							
No	582539	3782	3724540.08	1.02	1 (Ref.)	1 (Ref.)	1 (Ref.)
Yes	347516	2256	2221749.55	1.02	1.05 (1.00, 1.11)	1.05 (1.00, 1.11)	1.06 (1.01, 1.12)
**HDL**							
No	530770	3460	3393404.43	1.02	1 (Ref.)	1 (Ref.)	1 (Ref.)
Yes	399285	2578	2552885.2	1.01	1.04 (0.99, 1.09)	1.04 (0.99, 1.09)	1.04 (0.98, 1.09)
**Number of components**							
0	144848	939	927582.2	1.01	1 (Ref.)	1 (Ref.)	1 (Ref.)

1	226443	1458	1449657.91	1.01	1.06 (0.98, 1.15)	1.06 (0.98, 1.15)	1.06 (0.98, 1.15)
2	224973	1484	1438720.74	1.03	1.14 (1.05, 1.24)	1.14 (1.05, 1.24)	1.15 (1.05, 1.24)
3	182378	1115	1165700.31	0.96	1.10 (1.00, 1.20)	1.10 (1.00, 1.20)	1.11 (1.01, 1.21)
4	111414	754	710635.6	1.06	1.25 (1.13, 1.38)	1.25 (1.13, 1.38)	1.27 (1.15, 1.40)
5	39999	288	253992.87	1.13	1.37 (1.20, 1.57)	1.37 (1.20, 1.57)	1.41 (1.23, 1.61)

Abbreviations: Ref. = reference; HDL = high-density lipoprotein. * Model 1: adjusted for age. † Model 2: Model 1 + smoking, alcohol consumption, and physical activity. ‡ Model 3: Model 2 + duration of HRT, age at menarche, age at menopause, and income status. § Metabolic syndrome and components were defined from blood tests and anthropometric measurements from the 2009–2010 examinations: waist circumfer-ence ≥ 85 cm, systolic blood pressure ≥ 130 mmHg, diastolic blood pressure ≥ 85 mmHg or use of antihypertensive medications, fasting glucose ≥ 100 mg/dL or use of hypoglycemic agents, triglycerides ≥ 150 mg/dL or use of li-pid-lowering medications, HDL cholesterol < 50 mg/dL or use of lipid-lowering medications. The presence of three or more out of five components was regarded as metabolic syndrome.

Table S2. Risk of Invasive Breast Cancer According to Changes in Metabolic Syndrome and Its Components

		Number of Participants	Number of Breast cancers	Duration	Rate	Model 1[*]	Model 2[†]	Model 3[‡]
**Metabolic Syndrome Status[§]**								
No MetS–No MetS		431790	2784	2762581.59	1.01	1 (Ref.)	1 (Ref.)	1 (Ref.)
No MetS–MetS		164474	1097	1053379.27	1.04	1.12 (1.05, 1.20)	1.12 (1.05, 1.20)	1.12 (1.05, 1.21)
MetS–No MetS		79687	484	509303.6	0.95	1.03 (0.93, 1.13)	1.03 (0.93, 1.13)	1.04 (0.95, 1.15)
MetS–MetS		254104	1673	1621025.18	1.03	1.17 (1.10, 1.24)	1.17 (1.10, 1.24)	1.18 (1.11, 1.26)
**Metabolic Syndrome Components[§]**								
**Waist Circumference**	No–No	588701	3727	3764454.12	0.99	1 (Ref.)	1 (Ref.)	1 (Ref.)
(≥ 85 cm)	No–Yes	89075	613	570480.14	1.07	1.16 (1.06, 1.26)	1.16 (1.06, 1.26)	1.17 (1.08, 1.28)
	Yes–No	86298	535	551433.29	0.97	1.06 (0.97, 1.16)	1.06 (0.97, 1.16)	1.08 (0.99, 1.18)
	Yes–Yes	165981	1163	1059922.08	1.10	1.22 (1.14, 1.31)	1.22 (1.14, 1.31)	1.26 (1.17, 1.34)
**Fasting Glucose**	No–No	474211	3103	3038048.5	1.02	1 (Ref.)	1 (Ref.)	1 (Ref.)
(≥ 100 mg/dL)	No–Yes	122454	776	783584.85	0.99	1.00 (0.92, 1.08)	1.00 (0.92, 1.08)	1.01 (0.94, 1.10)
	Yes–No	110767	654	708623.82	0.92	0.92 (0.85, 1.01)	0.92 (0.85, 1.01)	0.93 (0.86, 1.02)
	Yes–Yes	222623	1505	1416032.45	1.06	1.11 (1.05, 1.19)	1.11 (1.05, 1.18)	1.13 (1.06, 1.20)
**Blood Pressure**	No–No	260587	1736	1669554.11	1.04	1 (Ref.)	1 (Ref.)	1 (Ref.)
(Systolic≥ 130 or	No–Yes	132764	766	850674.85	0.90	0.94 (0.86, 1.02)	0.94 (0.86, 1.02)	0.95 (0.87, 1.03)
diastolic ≥ 85 mmHg)	Yes–No	73625	461	471718.58	0.98	1.00 (0.90, 1.11)	1.00 (0.91, 1.11)	1.01 (0.92, 1.12)
	Yes–Yes	463079	3075	2954342.09	1.04	1.17 (1.10, 1.24)	1.17 (1.10, 1.24)	1.18 (1.11, 1.25)
**Triglycerides**	No–No	398941	2581	2548932.48	1.01	1 (Ref.)	1 (Ref.)	1 (Ref.)
(≥ 150 mg/dL)	No–Yes	183598	1201	1175607.59	1.02	1.06 (0.99, 1.14)	1.06 (0.99, 1.14)	1.06 (0.99, 1.14)
	Yes–No	91795	575	586557.29	0.98	1.01 (0.92, 1.11)	1.01 (0.92, 1.11)	1.03 (0.94, 1.13)
	Yes–Yes	255721	1681	1635192.26	1.03	1.10 (1.03, 1.17)	1.10 (1.03, 1.17)	1.10 (1.04, 1.17)
	No–No	328992	2158	2101628.01	1.03	1 (Ref.)	1 (Ref.)	1 (Ref.)
**HDL**	No–Yes	201778	1302	1291776.42	1.01	1.04 (0.97, 1.11)	1.04 (0.97, 1.11)	1.03 (0.96, 1.11)
(< 50mg/dL)	Yes–No	108086	664	691023.75	0.96	0.98 (0.90, 1.07)	0.98 (0.90, 1.07)	0.98 (0.90, 1.07)
	Yes–Yes	291199	1914	1861861.45	1.03	1.08 (1.02, 1.15)	1.08 (1.02, 1.15)	1.07 (1.01, 1.14)

Abbreviations: Ref. = reference; HDL = high-density lipoprotein. [*] Model 1: adjusted for age. [†] Model 2: Model 1 + smoking, alcohol consumption, and

physical activity. [‡] Model 3: Model 2 + duration of HRT, age at menarche, age at menopause, and income status. [§] Metabolic syndrome and components were defined from blood tests and anthropometric measurements from 2009–2010 and 2011–2012 examinations: waist circumference ≥ 85 cm, systolic blood pressure ≥ 130 mmHg, diastolic blood pressure ≥ 85 mmHg or use of antihyper-tensive medications, fasting glucose ≥ 100 mg/dL or use of hypoglycemic agents, triglycerides ≥ 150 mg/dL or use of lipid-lowering medications, HDL cholesterol < 50 mg/dL or use of lipid-lowering medications. The presence of three or more out of five components was regarded as metabolic syndrome.
